# Radiosensitization and growth inhibition of cancer cells mediated by an scFv antibody gene against DNA-PKcs in vitro and in vivo

**DOI:** 10.1186/1748-717X-5-70

**Published:** 2010-08-12

**Authors:** Li Du, Li-Jun Zhou, Xiu-Jie Pan, Yu-Xiao Wang, Qin-Zhi Xu, Zhi-Hua Yang, Yu Wang, Xiao-Dan Liu, Mao-Xiang Zhu, Ping-Kun Zhou

**Affiliations:** 1 Department of Radiation Toxicology and Oncology, Beijing Institute of Radiation Medicine, 27 Taiping Road, Haidian District, Beijing 100850, China; 2 The Centre of Clinical Laboratory, Navy General Hospital, PLA, Beijing 100037, China

## Abstract

**Background:**

Overexpression of DNA-dependent protein kinase catalytic subunit (DNA-PKcs) is commonly occurred in cancers and causes radioresistance and poor prognosis. In present study, the single-chain variable antibody fragments (scFv) targeting DNA-PKcs was developed for the application of radiosensitization in vitro and in vivo. A humanized semisynthetic scFv library and the phage-display antibodies technology were employed to screen DNA-PKcs scFv antibody.

**Methods:**

DNA-PKcs epitopes were predicted and cloned. A humanized semisynthetic scFv library and the phage-display antibodies technology were employed to screen DNA-PKcs scFv antibody. DNA damage repair was analyzed by comet assay and immunofluorescence detection of γH2AX foci. The radiosensitization in vivo was determined on Balb/c athymic mice transplanted tumours of HeLa cells.

**Results:**

Four epitopes of DNA-PKcs have been predicted and expressed as the antigens, and a specific human anti-DNA-PKcs scFv antibody gene, anti-DPK3-scFv, was obtained by screening the phage antibody library using the DNA-PKcs peptide DPK3. The specificity of anti-DPK3-scFv was verified, *in vitro*. Transfection of HeLa cells with the anti-DPK3-scFv gene resulted in an increased sensitivity to IR, decreased repair capability of DNA double-strand breaks (DSB) detected by comet assay and immunofluorescence detection of γH2AX foci. Moreover, the kinase activity of DNA-PKcs was inhibited by anti-DPK3-scFv, which was displayed by the decreased phosphorylation levels of its target Akt/S473 and the autophosphorylation of DNA-PKcs on S2056 induced by radiation. Measurement of the growth and apoptosis rates showed that anti-DPK3-scFv enhanced the sensitivity of tumours transplanted in Balb/c athymic mice to radiation therapy.

**Conclusion:**

The antiproliferation and radiosensitizing effects of anti-DPK3-scFv via targeting DNA-PKcs make it very appealing for the development as a novel biological radiosensitizer for cancer therapeutic potential.

## Background

Radiotherapy is one of the effective and common measures for cancer therapy. However, there are still some drawbacks which limit the clinic application of radiotherapy, *e.g*. severe side effects resulting from normal tissues damage and radiation tolerance of cancer cells [1]. DNA double-strand break (DSB) is a critical lesion induced by ionizing radiation (IR) [2], and the status of cellular DSB repair capability is closely related to the radiosensitivity and the outcome of radiotherapy[3,4]. DNA-dependent protein kinase catalytic subunit (DNA-PKcs) is a critical component in NHEJ pathway of DNA DSB repair [5], and it has a serine/threonine kinase activity to phosphorylate its downstream targets, such as Artemis, XRCC4, as well as autophosphorylation on its S2056 site [6,7]. Recent evidence indicates that DNA-PKcs is frequently overexpressed in various cancers, and increased expression or activity of DNA-PKcs is closely associated with metastasis, poor prognosis and radioresistance of cancers [1,8-13]. Depression of DNA-PKcs not only sensitizes cells to radiation, but also results in a decrease in cell growth rate and c-Myc protein levels [14]. Therefore, targeting DNA-PKcs has been promised as an effective approach for enhancing the efficiency of cancer radiation therapy [13-16].

Several chemical inhibitors of DNA-PKcs have been shown a radiosensitization effect in vitro, such as non-specific PI3K inhibitors (Wortmannin, LY294002) [17], DNA-PK inhibitors (OK-1035, NU7026) [18]. However, the relative low specificity and/or side effects to normal tissues have limited their clinical application. Due to their low immunogenicity in human being, humanized mAbs are becoming increasingly important biological measure of cancer therapy. Development of the humanized phage antibody library allows for screening single-chain variable antibody fragment (scFv). In essence, scFv is a small protein made up of both variable heavy and light chain domains coupled by a flexible peptide linker, and it is less immunogenic, of greater affinity, and more easily introduced into cells than antibodies produced by ordinary methods. Therefore, development of single-chain antibodies is a potential therapeutic strategy for cancer treatment. There is a report described the production and radiosensitizing effect in vitro of a scFv antibody against DNA-PKcs [19]. This scFv antibody was originally generated from a hybridoma cell line expressing the mAb 18-2 antibody of DNA-PKcs. However, it is necessary to expand this kind of study, especially to verify the efficacy and mechanisms of the radiosensitization of this kind of scFv molecules through the combined studies of cellular mechanistic experiments and the pre-clinical animal radiotherapy trial in vivo.

In this study, a specific anti-DNA-PKcs scFv antibody has been identified by screening a humanized phage library using purified DNA-PKcs epitopes. The gene encoding anti-DNA-PKcs-scFv was cloned and transfected into HeLa cells. HeLa cells expressing anti-DPK3-scFv displayed an increased radiosensitivity, decreased DNA-PKcs activity and deficient DSB repair. In addition, nude mouse xenograft tumours of HeLa cells expressing anti-DNA-PKcs-scFv became more sensitive to radiation therapy, indicating that anti-DNA-PKcs-scFv has the therapeutic potential. This anti-DNA-PKcs scFv provides a new tool for developing cancer therapeutic agent and the mechanistic study of DNA-PKcs in the cellular responses to radiation.

## Methods

### Cell culture

HeLa-DPK3-scFv and HeLa-pcDNA cell lines were generated from HeLa cells by transfecting with a DPK3-scFv expressing vector (pcDNA-DPK3-scFv) and the control vector (pcDNA3.1/Myc-His (-) B), respectively. The cells were grown in Dulbecco's modified Eagle's medium containing 10% fetal bovine serum, at 37°C in a humidified atmosphere of 5% CO_2_/95% air.

### Epitope production and scFv selection from phage-display library

The epitopes DNA-PKcs were predicted by a patent Biosun software, which was developed by Dr. Jian-Nan Feng from Beijing Institute of Basic Medicine, China, based on hydrophilicity, flexibility, antigenicity, charge distribution and other parameters. The sequence of peptide DPK3 with high antigenicity and no homology with other proteins was determined. DPK3 was amplified by RT-PCR, cloned into pET-22b (+), and transfected into *E. coli *BL21 (DE3) cells. The expressed DPK3 in *E. coli *was purified and refolded.

We employed a humanized semi-synthetic scFv library that was previously constructed in our laboratory. Screening of the phage-display antibody libraries was performed as previously described [20]. Briefly, the immunotubes coated with antigen DPK3 were incubated with the phage library (typically 10^13 ^cfu) at 37°C for 2 h. After washing with 0.05% Tween-PBS, the adherent phages were eluted using elution buffer (0.1 M HCl, pH 2.2 with solid glycine containing 0.1% BSA) and neutralized with 2 M Tris buffer. Eluted phages were used to infect *E. coli *XL1-Blue cells, and the Helper phage VCS-M13 (10^12 ^pfu) was then added and incubated at 30°C overnight. Phage preparation and screening was repeated 4 times. Individual ampicillin-resistant colonies (phage clones) were selected, and the supernatants of phage cultures were further obtained.

The positive anti-DNA-PKcs phage clones were determined by ELISA assay in the Microtiter plates (Nunc) coated with purified DPK3 as antigen or ovalbumin (OA) and ferritin (Fer) as negative controls. After incubation with the above phage clones suspension, the amount of bound phage was determined using HRP-labeled anti-M13 antibody (Amersham Biosciences, Piscataway, NJ) and developed by adding OPD (o-phenylenediamine). The reaction was monitored in a Spectra Max 340 ELISA reader (Molecular Devices, Sunnyville, CA) at 450 nm with a reference wavelength of 650 nm.

### Genetic fingerprint assay

The scFv DNA fragment of the selected phage clones with specific anti-DPK3 activity was amplified. PCR products were purified and digested with Mva I at 37°C for 2 h. The diversity of scFv was analyzed by PAGE followed by zymography and ethidium bromidine (EB) staining.

### Expression and identification of soluble anti-DPK3-scFv

Positive phage clones obtained from *E. coli *XL1-Blue were used to infect *E. coli *HB2151 non-suppressor bacterial strain to obtain soluble scFv. After overnight induction with 1 mM IPTG at 30°C, the antibody fragments were harvested from the supernatant and periplasmic space. ELISA was performed to screen for positive anti-DPK3 scFv fragments. Microtiter plate wells were coated with 50 μl DPK3 (10 μg/ml), using OA and Fer as negative controls, and incubated with 50 μl soluble anti-DPK3 scFv for 1 h at 37°C. Then anti-V5 antibody (R961-25, Invitrogen, Carlsbad, CA) was added for 1 h at 37°C. The amount of bound antibody was determined using HRP-labeled anti-Fab antibodies (I5260, Sigma) and developed by OPD.

### Sequencing of anti-DPK3-scFv and construction of eukaryotic expression vector

The scFv DNA fragment of the selected clones with specific anti-DPK3 activity was sequenced. scFv fragments were amplified and cloned into the eukaryotic expression vector pcDNA3.1/myc-His (-) B (Invitrogen). The vector was transfected into HeLa, and the stable cell lines were selected with G418.

### Irradiation and clonogenic survival

Cells were irradiated at room temperature using a ^60^Co γ-ray source at a dose rate of 1.6 Gy/min. Colony forming assays were performed immediately after irradiation by plating cells (3 × 10^2 ^to 1 × 10^4^) into 60-mm-diameter Petri dishes in triplicate. After 9 days of culture, cells were fixed with methanol, stained with Giemsa solution, and colonies (>50 cells) were counted and the survival curves were plotted.

### Comet assay and γH2AX foci detection

Comet assay and immunofluorescent γ-H2AX foci detection of DNA DSB were performed as previously described [21].

### Caspase-3/7 activity assay

Cells were collected and lysed with lysis buffer (50 mmol/L Tris-HCl, pH 7.5, 1% Nonidet P40, 0.5% sodium deoxycholate, 150 mmol/L NaCl, 1 piece of Protease inhibitor cocktail tablet in 50 ml solution). Cell lysates containing 25 μg of protein/well were incubated for 2 h at room temperature with Apo-ONE^® ^Caspase-3/7 Reagent (G7790, Promega, Madison, WI). The fluorescence of each well was measured using a 96-well fluorescence plate reader at an excitation wavelength of 485 nm and an emission wavelength of 538 nm. Data were expressed as arbitrary units of fluorescence per microgram of protein (F/mg protein). The average level of F/mg protein was calculated from three independent experiments.

### DNA-PKcs activity detection

DNA-PKcs activity was detected using the Signa-TECT^® ^DNA-Dependent Protein Kinase Assay System (Promega), in which a DNA-PK biotinylated p53-derived peptide acts as the substrate of DNA-PKcs. The nuclear proteins were extracted from HeLa cells, HeLa-pcDNA cells or HeLa-DPK3-scFv cells 30 min after 4 Gy irradiation or control. Following the manufacturer's protocol, reaction mixtures (25 μl) contained 6 μg of nuclear proteins from HeLa cells, HeLa-pcDNA cells, or HeLa-DPK3-scFv cells, DNA-PK activation buffer, reaction buffer, and 0.5 mCi [γ-^32^P]ATP. Samples were incubated at 30°C for 10 min. Termination buffer was then added and 10 μl of each reaction mixture was spotted onto SAM2^® ^capture membrane. After washing with 2 M NaCl, membranes were dried and subjected to the quantification of the activities of incorporated ^32^P-phosphorylated substrate by Molecular Dynamics Phosphoimager System.

### Immunohybridization analysis

The antibodies used in this study were purchased commercially: anti-Akt (#9271, Cell signal, Danvers, MA), anti-phospho-Akt (Ser473, #9271, Cell signal, Danvers), anti DNA-PKcs (phospho S2056) antibody (ab18192, Abcam, MA), anti-DNA-PKcs antibody (H-163, Santa Cruz, CA), anti-β-actin (I-19-R, Santa Cruz). Immunohybridization analysis was performed as previously described [22].

### Antitumour activity in vivo

The female athymic mice (Balb/c, nu/nu) were obtained from the Laboratory Animal Center (Beijing, China). The Institutional Animal Licensing Committee has approved the animal experiment undertaken, and the research protocol was in accordance with the institutional guidelines of the Animal Care and Use Committee. HeLa-pcDNA and HeLa-DPK3-scFv cells of 10^7 ^cells resuspended in 200 μl of DMEM were subcutaneously injected in the lateral back region. Once tumours had reached a volume of about 300 mm^3^, mice were randomly placed into the non-irradiation (control) or irradiation group (+ IR). Tumours in treated mice were irradiated 2 Gy once every other day with total of 10 Gy, while the rest of the body was shielded with lead brick. Serial measurements of tumour diameter were made with calipers after each irradiation. Tumour volume = width^2 ^× length/2.

### Immunohistochemistry and TUNEL assay

Tumour tissue was dissected from euthanized nude mice, immediately fixed in formalin and embedded in paraffin. Sections were de-paraffinized by heating to 60°C followed by xylene immersion and re-hydrated with sequential ethanol submersion. Endogenous catalase activity was eliminated with 3% H_2_O_2 _and sections were blocked and incubated with anti-cleaved caspase-3 antibody (9661, Cell Signaling Technology) at a dilution of 1:50 at 4°C overnight and, subsequently, developed with Streptavidin-Biotin Complex (SABC). The terminal deoxynucleotidyl transferase (TdT)-mediated dUTP nick end labeling (TUNEL) assay was also performed using the In Situ Cell Death Detection Kit, POD (11684817910, Roche, Indianapolis, IN). Positive staining was analyzed with the Mias 2000 Imaging Analysis System.

### Statistics

Data are presented as mean ± SD. Statistical analyses of data were carried out using Student's *t*-test. *P *< 0.05 was considered to be statistical significant. One-way analysis of variance (ANOVA) was used to compare the groups of the in vivo animal radiotherapy experiments followed by the Student-Newman-Keuls method for multiple comparisons.

## Results

### Identification of antigen fragment of DNA-PKcs

**Figure 1:**
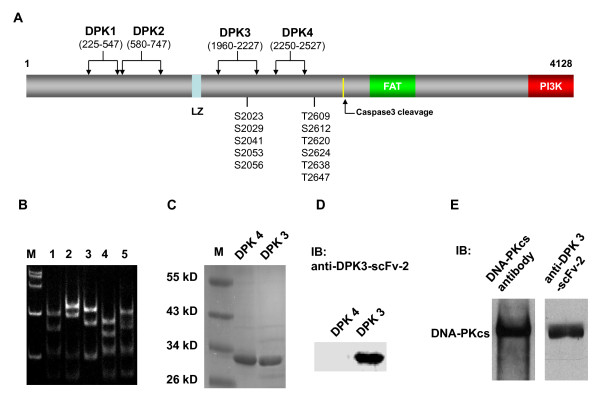
**Screening and characterization of positive phage clones of anti-DNA-PKcs segments scFv**. (A) Conserved functional domains of DNA-PKcs and the location of segments identified with epitopes predicted in DNA-PKcs. DPK1, DPK2 are located at the N-terminus of DNA-PKcs, and DPK3 and DPK4 are located between the leucine zipper (LZ) and T2609 autophosphorylation cluster. The DPK3 segment includes Ser2056 autophosphorylation cluster. (B) PAGE electrophoresis patterns of the Mva I-digested positive anti-DPK3-scFv clones. (C) Coomassie brilliant blue stained gel of the SDS-PAGE analysis of 10 μg purified DPK3 (~30 kD) and DPK4 (~32 kD) segments. (D) Immunohybridization (western blotting) analysis of purified DPK3 (30 kD) and DPK4 (32 kD) segments using anti-DPK3-scFv-2 antibody. (E) Immunohybridization analysis of DNA-PKcs in HeLa cell protein lysate using anti-DPK3-scFv-2 and anti-DNA-PKcs antibodies.

DNA-PKcs is approximately 470 kD consisting of 4128 amino acids. Its C-terminus contains a serine/threonine protein kinase domain (3719- 4128) [23]. For screening the specific anti-DNA-PKcs scFv from the phage library, 6 epitopes of DNA-PKcs have been predicted as the antigens. DPK5 and DPK6 peptides are located in the focal adhesion targeting (FAT) domain, which is homologous with other members of the PIKK family [24], so they were rejected. The DPK1 and DPK2 peptides are located in the N-terminus of DNA-PKcs, and DPK3 and DPK4 between the leucine zipper and the autophosphorylation cluster (Figure 1A). The cDNA encoding these peptides were cloned, and the peptides were expressed in and purified from the infected *E coli *cells. Upon inclusion body protein degeneration and refolding, we have finally successfully obtained the soluble DPK3 peptide of ~30 kD with the purity of >95%.

### Identification of the positive phage clones of scFv antibody

**Figure 2:**
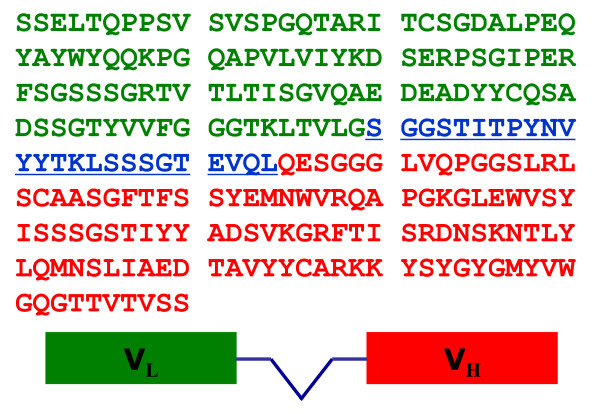
**The Sequence of the anti-DPK2-scFv-2**. The green colour and the red colour is the sequence of variable light chain (V_L_) and variable heavy (V_H_), respectively. The blue colour is the linker.

Following 4 rounds of selection using a human phage-display antibody library, 56 clones bound to DPK3 were obtained, and 26 positive clones were confirmed by ELISA. Clones were considered positive only if they did not bind to negative ovalbumin and ferritin control and showed >3-fold increase in OD = 450nm. The Mva I-restriction maps of five clones were shown in Figure 1B. Non-suppressive *E coli *HB2151 bacteria cells were transduced with positive clones to obtain soluble scFv. Competition test of ELISA indicated that the soluble anti-DPK3-scFv-2 in Figure 1B showed a highest specificity of antibody. The sequence data shown in Figure 2 demonstrate that its variable heavy (V_H_) and light chain (V_L_) belong to VH4 sub-group and VL2 sub-group, respectively. The specificity of anti-DPK3-scFv encoded by DPK3-scFv-2 gene was further validated, which immunohybridizes with the purified DPK3, but not DPK4 (Figure 1C &1D). In addition, immunohybridization analysis of total extract of HeLa cells using anti-DPK3-scFv-2 also showed an unique immunobinding band with DNA-PKcs as comparable with the commercial rabbit polyclonal anti-DNA-PKcs antibody (Figure 1E), suggesting no cross immunoblotting reactivity with HeLa cell extracts. Therefore, anti-DPK3-scFv-2/gene was chosen for further studies.

### Expression of anti-DPK3-scFv sensitizes HeLa cells to radiation

**Figure 3:**
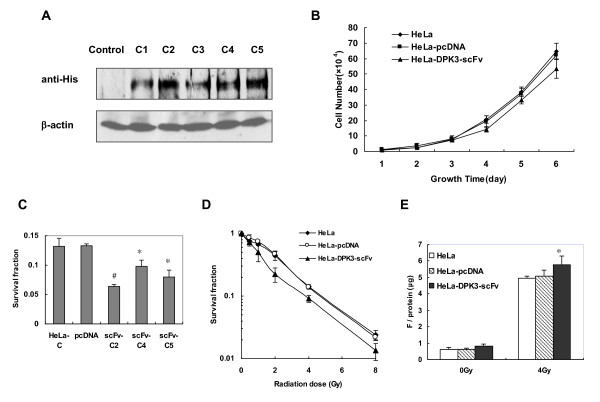
**Effect of anti-DPK3-scFv on cellular survival**. (A) Immunohybridization analysis of anti-DPK3-scFv expression in HeLa cell clones (C1 - C5) stably transfected with His-anti-DPK3-scFv-2 using an anti-His antibody. (B) Growth rates for HeLa, HeLa-pcDNA and DPK3-scFv-2-transfected HeLa cells (HeLa-DPK3-scFv) under normal growing condition. (C) Clonogenic assays of cells survivals for HeLa, HeLa-pcDNA and the clones (scFv-C3, C4, C5) of DPK3-scFv-2-transfected HeLa cells after 4 Gy γ-ray irradiation. * *P *< 0.05, ^#^*P *< 0.01 as compared with control HeLa-pcDNA cells. (D) Cell survival curves of HeLa, HeLa-pcDNA and HeLa-DPK3-scFv cells (clone 2) post-irradiation. (E) Caspase-3 activity assays of HeLa, HeLa-pcDNA and HeLa-DPK3-scFv cells post-4 Gy irradiation. * *P *< 0.05 as compared with control HeLa or HeLa-pcDNA cells.

HeLa-DPK3-scFv cell clones (C1 to C5) were generated from HeLa cells stably transfected with His-tagged anti-DPK3-scFv-2 gene and the expression level of anti-DPK3-scFv in each clone was shown in Figure 3A. The growth rate of the anti-DPK3-scFv-transfected cells (HeLa-DPK3-scFv) is lightly slower as compared with HeLa cells or the cells transfected with the mock vector (HeLa-pcDNA) (Figure 3B), but this growth difference is not statistically significant. There is no different in cell cycle distribution among these three cell lines under normal growing conditions (data not shown). Survival assay of 4 Gy-irradiated cells determined that anti-DPK3-scFv-transfected cells were more sensitive to γ-ray compared to control cells, with the HeLa-DPK3-scFv-c2 (clone 2) being the most sensitive clone (Figure 3C). Clonogenic assay was further performed for the cells after 0 - 8 Gy γ-ray irradiation. Cell survival curves demonstrate an increased radiosensitivity for the HeLa-DPK3-scFv cells (clone 2) (Figure 3D). There was approximately a 20% dose reduction required for the same level of survival.

As shown in Figure 3E, caspase-3 activity was increased in all cell lines post-irradiation, while the activity was relative higher in HeLa-DPK3-scFv cells compared to HeLa and HeLa-pcDNA cells, implying that anti-DPK3-scFv antibody increases the induction of apoptosis by radiation.

### Anti-DPK3-scFv decreases DNA-PKcs activity responding to radiation

**Figure 4:**
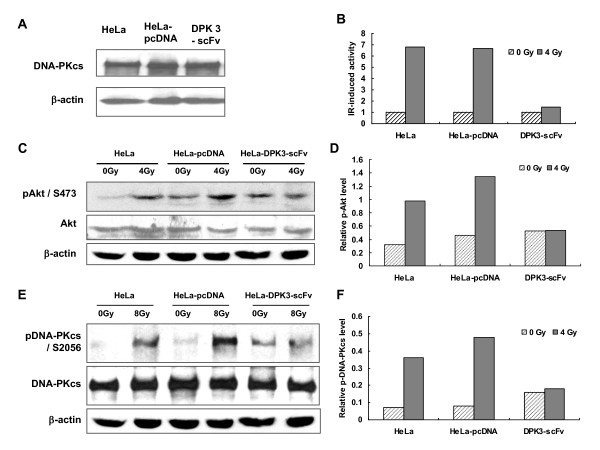
**The effects of anti-DPK3-scFv on DNA-PKcs activity**. (A) Immunohybridization analysis of total DNA-PKcs protein level in HeLa, HeLa-pcDNA and HeLa-DPK3-scFv cells. (B) In vitro detection of 4 Gy-induced DNA-PKcs activity using the Signa-TECT^® ^DNA-Dependent Protein Kinase Assay System. Immunoblotting pattern (C) and relative p-Akt level (the ratio of p-Akt/Akt signals) (D) of Akt, p-Akt/S473 in HeLa, HeLa-pcDNA and HeLa-DPK3-scFv cells post-4 Gy irradiation. Immunoblotting pattern (E) and relative p-DNA-PKcs level (the ratio of p-DNA-PKcs/DNA-PKcs signals) (F) of DNA-PKcs, p-DNA-PKcs/S2056 in HeLa, HeLa-pcDNA and HeLa-DPK3-scFv cells post-8 Gy irradiation.

The expression of DNA-PKcs in anti-DPK3-scFv expressing cells was determined by Western blot analysis. Result shows that anti-DPK3-scFv does not affect the expression of DNA-PKcs (Figure 4A). The effect of anti-DPK3-scFv on DNA-PK activity in vitro was assayed, by monitoring the ability of the nuclear extract from the treated cells to phosphorylate the DNA-PK-specific target p53 peptide with [γ-^32^P] ATP. As shown in Figure 4B, the activity of DNA-PK induced by 4 Gy irradiation was greatly abolished in the HeLa-DPK3-scFv cells as compared with HeLa cells and HeLa-pcDNA cells. Akt is an anti-apoptotic factor that is phosphorylated at Ser-473 by the activated DNA-PKcs [25]. After 4Gy γ-irradiation, the phosphorylated Akt/S473 increased significantly in the HeLa and HeLa-pcDNA cells, but scarcely in HeLa-DPK3-scFv cells (Figure 4C &4D). DNA-PKcs pS2056 is an autophosphorylated site of DNA-PKcs, and it is included in the fragment of DPK3 (AA1960-2227). After 8Gy γ-irradiation, the phosphorylated DNA-PKcs at S2056 remarkably increased in the control HeLa and HeLa-pcDNA cells. However, this radiation-induced autophosphorylation was largely abrogated in HeLa-DPK3-scFv cells (Figure 4E &4F). These results indicate that anti-DPK3-scFv decreases the activity of DNA-PKcs in response to radiation.

### Anti-DPK3-scFv decreases DNA double-strand break repair (DSBR)

**Figure 5:**
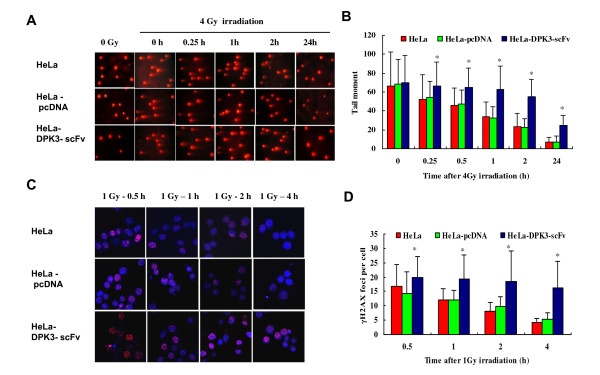
**The effects of anti-DPK3-scFv on DNA double-strand breaks repair**. (A) The comet images detected by neutral single-cell gel electrophoresis (SCGE). (B) DNA DSB and repair expressed as the comet tail moment of SCGE assays in HeLa, HeLa-pcDNA and HeLa-DPK3-scFv cells at given times post-4 Gy irradiation. The data are means and standard deviation from three independent experiments. * *P *< 0.05 as compared with parental HeLa cells and control cells at the same time point. (C) Immunofluorescence detection of γ-H2AX foci in the cells 0.5 h to 4 h post-irradiation. (D) The residual number of γ-H2AX foci per nucleus in the cells 0.5 h to 4 h post-irradiation. * *P *< 0.05 as compared with parental HeLa cells and control cells at the same time point.

Neutral comet assay was performed to determine the efficiency of DSBR. The comet tail of HeLa-DPK3-scF cells was much longer than that of HeLa and HeLa-pcDNA cells after 4Gy γ-ray radiation (Figure 5A). Residual DNA damage in HeLa-DPK3-scFv cells was significantly higher than that of HeLa and HeLa-pcDNA cells 0.25 h to 4 h of post-irradiation (Figure 5B).

The kinetics of γ-H2AX foci induction and elimination was measured in 1Gy γ-ray irradiated cells by immunofluorescence assay (Figure 5C &5D). Results demonstrated that the residual number of γ-H2AX foci per nucleus in HeLa-DPK3-scFv cells was significantly higher than that of HeLa and HeLa-pcDNA cells 0.5 h to 4 h post-irradiation. These data further indicate that anti-DPK3-scFv results in deficiency of DNA DSB repair in HeLa-DPK3-scF cells.

### Anti-DPK3-scFv sensitizes tumours to radiotherapy in mice

**Figure 6:**
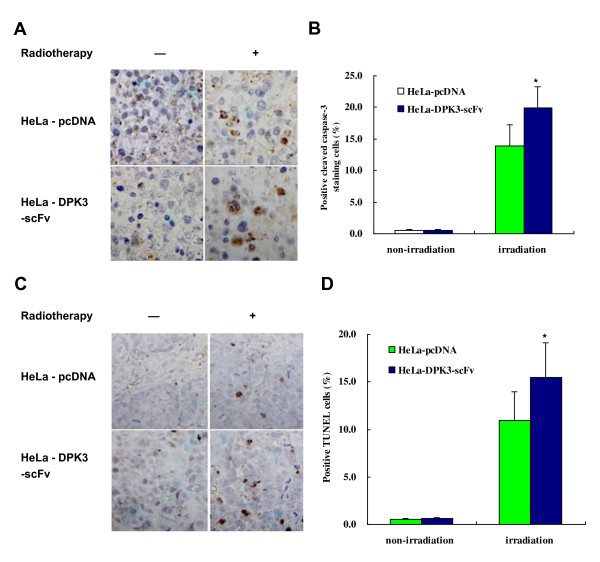
**The effect of anti-DPK3-scFv on apoptosis induction of the xenografted cancer cells in nude mice by irradiation**. (A) Immunohistochemistry staining of cleaved caspase-3 in the xenografted tumour tissue sections either non-irradiated or 3 days after the radiotherapy. (B) Expression level of cleaved Caspase-3 in xenografted tumour tissue sections. (C) TUNEL staining of the xenografted tumour sections either non-irradiated or 3 days after the radiotherapy. (D) Apoptosis induction measured from the TUNEL staining of xenografted tumour tissue sections. Expression levels of the detected proteins were expressed as the percentage of positive staining cells. 100 cells were scored for each staining. * *P *< 0.05 as compared with the tissues of HeLa -pcDNA xenografted tumours.

To investigate the potential for combined anti-DPK3-scFv gene therapy and irradiation therapy, *in vivo*, HeLa-pcDNA and HeLa-DPK3-scFv cells were transplanted into athymic mice. Xenografts were irradiated as described in materials and methods. Result shows that anti-DPK3-scFv largely attenuated the growth rate of the tumours generated from HeLa-DPK3-scFv cells after the radiotherapy (Table 1). Detection of cleaved caspase-3 (Figure 6A &6B) and TUNEL assay (Figure 6C &6D) in tumour tissue sections shows that the radiation-induced apoptosis in HeLa-DPK3-scFv xenografts was much higher than HeLa-pcDNA xenografts. These data demonstrate that anti-DPK3-scFv enhances the sensitivity of tumours to radiotherapy.

**Table 1:** Sensitization of DPK3-scFv on tumours in nude mice to radiotherapy.*

Treatment groups + IR (irradiation)	**Tumour volume (mm**^**3**^**)** (The time from the beginning of radiotherapy, day)					
	
	0	2	4	6	8	10
bpcDNA	348.9 ± 183.0	487.5 ± 223.0	726.3 ± 252.1	808.7 ± 265.9	829.7 ± 279.9	929.2 ± 338.7
	(n = 10) ^#^	(n = 10)	(n = 10)	(n = 9)	(n = 9)	(n = 6)
pcDNA + IR	317.8 ± 127.2	520.4 ± 218.5	720.8 ± 361.4	840.4 ± 381.8	776.5 ± 193.5	757.1 ± 266.7
	(n = 11)	(n = 11)	(n = 11)	(n = 11)	(n = 11)	(n = 10)
DPK3-scFv	391.4 ± 213.6	531.7 ± 258.5	730.6 ± 394.4	831.3 ± 399.7	861.2 ± 331.1	938.0 ± 273.7
	(n = 13)	(n = 13)	(n = 13)	(n = 11)	(n = 11)	(n = 10)
DPK3-scFv + IR	394.8 ± 216.9	581.6 ± 408.5	621.4 ± 323.2	613.6 ± 282.9	521.9 ± 266.1^$^	572.7 ± 363.9^@^
	(n = 10)	(n = 10)	(n = 10)	(n = 10)	(n = 9)	(n = 8)

* HeLa-pcDNA (control) and HeLa-DPK3-scFv cells were transplanted into athymic nude mice. Radiotherapy was performed with 60 Co γ-rays as described in the materials and methods, and tumour sizes were measured after each irradiation (+ IR) or at the same time for the non-irradiated tumours. The data are the means ± SD. One-way analysis of variance (ANOVA) was used for the statistical significant analysis.

^# ^The animal number (n). There were 10 to 13 mice for different group at the beginning of the radiotherapy, and there occurred animal death from the sixth day after the beginning of radiotherapy.

^$ ^*p *= 0.02, 0.044, 0.035 as compared with the group pcDNA, pcDNA + IR and DPK-scFv at the same day (day 8), respectively.

^@ ^*p *= 0.039, 0.017 as compared with the group pcDNA and DPK-scFv at the same day (day 10), respectively.

## Discussion

In this study, a specific scFv antibody against DNA-PKcs (anti-DPK3-scFv) has been identified by screening a phage library using a purified DNA-PKcs fragment (DPK3, AA1960 - 2227) as antigen. Expression of anti-DPK3-scFv in HeLa cells increased the radiosensitivity, and resulted in a deficient of DNA DSB repair. In addition, tumour xenografts generated from HeLa cells expressing anti-DPK3-scFv exhibited increased radiosensitivity. These observations demonstrated that anti-DPK3-scFv is a potential effective radiosensitizer for cancer radiotherapy.

Antibodies have been proven effective in inhibiting the function of proteins by blocking the activity domain or critical protein interaction sites. There have been numerous antibody drugs undergoing pre-clinical and clinical trials, especially in the field of oncology [26]. In this study, epitope prediction has been done prior to screening phage display for anti-DNA-PKcs-scFv. Interestingly, the DPK3 segment (AA1960-2227), that was predicted a high antigenicity in this study, harbors the antigen epitope of the anti-DNA-PKcs-scFv 18-2 antibody generated by Li et al [19]. In their study, scFv 18-2 was reversely amplified from the mRNA extracted from the hybridoma cells expressing mAb 18-2 antibody, and its epitope was mapped to a fragment spanning DNA-PKcs residues 1734-2228. The scFv18-2 was demonstrated a similar inhibiting effect as the parental mAb on the kinase and DNA end joining activities of DNA-PKcs in cell-free systems. Microinjection of purified scFv18-2 protein sensitized human skin melanoma SK-MEL-28 cells to radiation and decreased DNA repair capability. Both the scFv antibodies described in the study and in previous report [19] verified that scFv antibody against DNA-PKcs is an appealing biological measure of radiosensitization. Differently, anti DPK3-scFv was obtained by screening a human phage-display library, so it has lower immunogenicity than antibodies generated through other methods. The sequence and molecular structure of DPK3-scFv are clear, and it can be produced in infinity. In addition, soluble anti-DPK3-svFv was shown to have high specificity for DNA-PKcs *in vitro*. In this study, expression of anti-DPK3-scFv in HeLa cells increased radiosensitivity, and inhibited DSBR. In addition, DNA-PKcs associated AKT phosphorylation at S473 and its autophosphorylation at S2056 induced by ionizing radiation was inhibited, and apoptosis was increased in cells expressing anti-DPK3-scFv. Finally, xenografts tumours expressing anti-DPK3-scFv exhibited increased radiosensitivity. Collectively, these data indicate that anti-DPK3-scFv owns the features of a biological radiosensitizer by targeting DNA-PKcs.

It is likely that the extent of radiosensitization by anti-DPK3-scFv is relative modest as compared with the DNA-PKcs chemical inhibitors or knockout of DNA-PKcs. Unlike the chemical inhibitors of DNA-PKcs, anti-DPK3-scFv does not directly target the C-terminal conserved kinase domain. As a gene product, DPK3-scFv approach may have also some limitations for the application on cancers radiosensitization. The biological activity of DPK3-scFv is largely dependent on the expression level, half-life time, and its accessible to the target protein. In addition, chemical modification of the epitope may also affect on the binding of DPK3-scFv with DNA-PKcs.

The phosphorylation of DNA-PKcs is necessary for DNA double-strand break (DSB) repair[27]. A phosphorylation cluster is located in the epitope DPK3 (Figure 1A) or the binding domain of DPK3-scFv, it is reasonable that DPK3-scFv can directly interfere the phosphorylations of DNA-PKcs, including site of S2056, which, in some extent, results in a deficient of DSB repair. Although the phosphorylation of epitope may affect the binding of DPK3-scFV with DNA-PKcs, DPK3-scFv can actually bind to the non-phosphorylated epitope/DNA-PKcs before the induction of DNA damage by IR, which result in an interference/blockage on the IR-induced phosphorylation of DNA-PKcs. This mechanistic action is unique for DPK3-scFv. Boskovic et al have shown that upon incubation with DNA, DNA-PKcs undergoes a conformational change that activates its kinase activity [28]. Anti-DPK3-scFv does not affect the total expression level of DNA-PKcs protein, so the mechanism of inhibition might involve either hindrance of some essential interaction sites of DNA-PKcs with its functional counterparts or blockage of a conformational change required for progression of the end joining reaction. Actually, DPK3 contains the autophosphorylation cluster around S2056, which has been proven to be associated with DSBR [7]. Therefore, the binding of DPK3-scFV with DNA-PKcs may competitively block the radiation-induced autophosphorylation DNA-PKcs on S2056. As the detail function of this domain is unclear, anti-DPK3-scFv would be also a good tool for future mechanism study of DNA-PKcs function.

## Conclusions

By using a human semisynthetic scFv library and the phage-display antibodies technology, we have obtained a DNA-PKcs specific scFv antibody, and which was shown to sensitize cancer cells to ionizing radiation in vitro and in vivo. Radiosensitization by this scFv antibody is association with the decrease of DSBR capability and partial inhibition of DNA-PKcs kinase activity. These data suggest that anti-DNA-PKcs scFv antibody provides a new strategy to improve therapeutic gain for radiation therapy.

## Competing interests

The authors declare that they have no competing interests.

## Authors' contributions

LD and LJZ carried out most of the study and participated in its design. XJP participated the experiments of DNA-PKcs expression and detection of its target phosphorylation. YXW has participated the screening of scFv antibody. QZX and MXZ participated the study design and data discussion. ZHY participated the animal study. YW participated the radiosensitivity analysis and in vitro analysis of DNA-PKcs activity. XDL have done the transfection of scFv antibody gene and cell clone identification. PKZ jointly conceived of the study, and coordination, participated in its design and drafted the manuscript. All authors read and approved the final manuscript.
